# Impact of Dietary Antioxidant Supplements on Atrophic Lesion Progression in Stargardt Disease: A Retrospective Observational Study

**DOI:** 10.1155/joph/5231278

**Published:** 2025-08-28

**Authors:** Christopher A. Turski, Michalis Georgiou, Cesar Estrada Puente, Kubra Sarici, Xiao Zhou, Ramiro S. Maldonado

**Affiliations:** Department of Ophthalmology, Duke University, Durham, North Carolina, USA

**Keywords:** ABCA4, antioxidants, fundus autofluorescence, Stargardt disease, supplements, therapy

## Abstract

**Purpose:** To investigate whether supplementation with dietary antioxidants has an effect on the yearly progression rate of atrophic lesions in autosomal recessive Stargardt disease (STGD1), as derived from fundus autofluorescence (FAF).

**Methods:** Retrospective study of patients with molecularly confirmed STGD1 aged ≥ 6 years at baseline and presence of an atrophic lesion of ≥ 250 μm in diameter, who underwent FAF imaging between 01/01/2010 and 10/31/2023. Patients were grouped into supplement takers and nontakers based on the daily intake of lutein, zeaxanthin, saffron, and N-acetylcysteine. Baseline and follow-up FAF images were graded by two readers. Variables assessed included total area of decreased FAF (DAF) and effective lesion size of total DAF, calculated as a square root transformation. Annual atrophy growth rates were calculated for both subgroups and compared.

**Results:** A total of 53 eyes of 53 patients were enrolled. Thirty-three patients were categorized as supplement takers (mean age 34 ± 20.3 years, 57.6% female) and 20 patients as nontakers (mean age 29.5 ± 16.6 years, 65% female). Mean growth rates of DAF were 0.61 ± 0.72 mm^2^/year for supplement takers and 0.49 ± 0.55 mm^2^/year for nontakers (*p*=0.9). The mean observation period was 2 years (range 0.5–5.6) and 4.5 years (range 1–10.3), for supplement takers and for nontakers, respectively.

**Conclusion:** Supplementation with dietary antioxidants did not result in a slower progression rate of DAF lesions in STGD1. Further investigation with randomized trials is needed for evidence-based use of antioxidant supplements for the disease.

## 1. Introduction

Autosomal recessive Stargardt disease (STGD1) is the most prevalent inherited macular dystrophy, typically characterized by central macular atrophy and yellow-white flecks at the level of the retinal pigment epithelium (RPE) at the posterior pole [1–3]. Mutations in the ATP-binding cassette, subfamily A, member 4 (*ABCA4*) gene, which encodes a transmembrane rim protein in the outer segment discs of photoreceptors, underlie STGD1 [4, 5]. Transport of retinoids from photoreceptors to the RPE is affected, resulting in increased accumulation of lipofuscin fluorophores in the RPE, in particular N-retinylidene-N-retinylethanolamine (A2E) [6, 7]. Over time, increasing levels of A2E are believed to induce phototoxicity with oxidative stress, resulting in photoreceptor degeneration, RPE dysfunction, and RPE loss [8–10].

Since oxidative stress is an important causative factor for retinal degenerative diseases, supplementation with antioxidants may have protective effects [11]. Lutein and zeaxanthin are xanthophyll carotenoids that have been shown to reduce accumulation of bisretinoids such as A2E in the retina and subsequently protect the eye from oxidative damage and enhance visual performance [11, 12]. Saffron contains biologically high concentrations of the carotenoids crocin and crocetin, yielding high antioxidant potential [13]. Focal electroretinogram (fERG) outcomes have indicated a potential for saffron to prevent progression of central retinal degeneration in STGD1 [14]. In addition, oral N-acetylcysteine (NAC), a derivative of L-cysteine that neutralizes reactive oxygen, has been shown to reduce oxidative damage and promote maintenance of function and survival of cones in a mouse model of retinitis pigmentosa (RP) and may improve cone function in RP patients [15, 16]. Furthermore, it has been described that supplementation with docosahexaenoic acid (DHA), a type of omega-3 fatty acid with antioxidant properties, may lead to a mild improvement in both visual acuity and multifocal electroretinography (mfERG) outcomes in patients affected with STGD1 [17].

Fundus autofluorescence (FAF) imaging, which uses autofluorescent properties of lipofuscin fluorophores, provides valuable information on the distribution of lipofuscin in the RPE [18]. Furthermore, FAF imaging allows reliable quantification of the progression rate of RPE atrophy as defined by the area of decreased FAF (DAF) [19]. Several studies have been conducted to report the annual growth rate of atrophic lesions in STGD1, ranging from 0.06 mm^2^/year to 5.27 mm^2^/year [20–26]. However, the impact of dietary antioxidants on the progression of these lesions in STGD1 has so far not been assessed.

In this study, we study the effects of dietary antioxidant supplements on the yearly progression rate of atrophic lesions in STGD1 as derived from FAF imaging in a large retrospective series.

## 2. Methods

The study protocol adhered to the tenets of the Declaration of Helsinki and received approval from the Institutional Review Board (IRB) and was conducted at the Department of Ophthalmology at Duke University in Durham, USA.

### 2.1. Patient Identification

The data exploration tool ‘SlicerDicer' was employed in the Duke Electronic Health Record (EHR) System using the search term ‘Stargardt disease' to identify patients who underwent baseline and follow-up FAF imaging between January 1, 2010, and October 31, 2023. Inclusion criteria were age ≥ 6 years at baseline with at least two pathogenic mutations in the *ABCA4* gene and presence of an atrophic lesion of ≥ 250 μm in diameter. Patients needed to follow up for at least two examinations over a period of at least 6 months. Exclusion criteria included the presence of ocular disease, such as choroidal neovascularization (CNV), glaucoma, or diabetic retinopathy, in both eye and the presence of poor-quality images or incomplete chart data/missing imaging.

### 2.2. Clinical Assessment

One eye of each patient was included in the study. The eye providing the higher quality FAF image was selected for recruitment in the study; however, where there was no discrepancy between fellow eyes in terms of FAF image quality, the right eye was arbitrarily selected as the study eye.

Medical records of each participant were reviewed for age, gender, race, number of pathogenic mutations in the *ABCA4* gene, best-corrected visual acuity (BCVA), bodyweight, and intake of dietary antioxidant supplements at the baseline visit and follow-up visits. Supplement intake had been universally recommended to patients by a single provider, and only some patients reported taking them. Supplement intake was not adjusted based on the patient's bodyweight.

Patients were grouped into supplement takers and nontakers based on the daily intake of at least one of lutein, zeaxanthin, saffron, and N-acetylcysteine.

### 2.3. Imaging Analysis

FAF images were obtained, after pupil dilation, using a Spectralis SD-OCT device (Heidelberg Engineering, Heidelberg, Germany), with excitation light, 488 nm; barrier filter, 500 nm; and field of view of 30° × 30° or 55° × 55°, centered on the macula.

**Figure 1:**
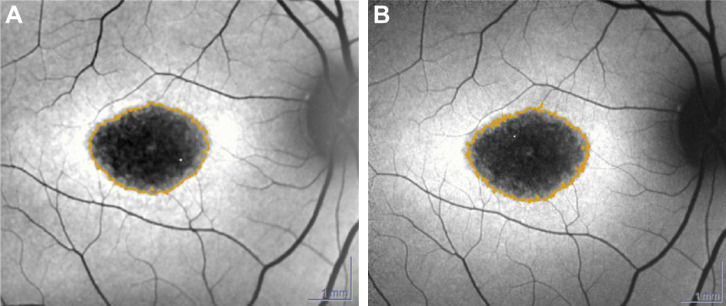
DAF quantification. (A) Lesion of DAF (4.391 mm^2^) at baseline in a supplement taker (patient 29) (taking lutein, zeaxanthin, saffron, and NAC). (B) Lesion in the same eye after 15 months enlarged to 4.823 mm^2^. Abbreviation: DAF = decreased autofluorescence.

FAF images at baseline and follow-up visits were graded for qualitative and quantitative features independently by two masked graders (CAT and CEP). Qualitative grading parameters at baseline included the absence or the presence of pisciform flecks, single or multiple atrophic lesions, foveal or nonfoveal lesions, presence of peripapillary atrophy, and the presence of increased FAF at the edge of a lesion DAF. In cases of discrepancy, a third more senior grader (KS) evaluated the images. Quantitative variables assessed included total DAF area and effective lesion size of total DAF, calculated as square root transformation (SQRT) [24]. Areas in which DAF was close (> 90% of darkness) to the level of vessels and the optic disc were defined as definitely DAF (DDAF), and the areas of DAF between 50% and 90% of darkness were defined as questionably DAF (QDAF) [23]. The total area of DAF (DDAF plus QDAF) at baseline and follow-up visits was semiautomatically outlined and quantified using the RegionFinder module of Heidelberg Eye Explorer (Figure 1) [27]. The final value was defined as the average of the two measurements between the graders, provided that the two measurements did not differ by > 0.3 mm^2^. If the lesion size deviated by > 0.3 mm^2^, a third masked grader (KS) additionally performed the measurement [28]. The final value was then calculated by averaging the third grader's measurement along with the closer of the two other grader measurements [29]. The rate of yearly progression (mm^2^/year and mm/year) was calculated for each individual subgroup of patients [24].

### 2.4. Statistical Analysis

Reliability and repeatability of the quantitative measurements were calculated as the average measure intraclass correlation coefficient based on a 2-way mixed-effects model, absolute agreement, and Bland–Altman analysis. BCVA at the baseline and final follow-up visits was recorded as Snellen visual acuity and converted to approximate Early Treatment Diabetic Retinopathy Study (ETDRS) letter equivalents for analysis. The Shapiro–Wilk test was used to assess the normality of the data. Wilcoxon signed-rank tests were used to compare mean BCVA in each subgroup at baseline and the final follow-up visit. Visual acuity reduction was calculated as the difference between the ETDRS letter score at baseline and the final follow-up visit.

Baseline lesion size and progression rates in groups of supplement takers and nontakers were compared by Mann–Whitney *U* tests. Chi-Square (*χ*^2^) tests were applied to analyze qualitative FAF parameters between subgroups at baseline. Statistical analyses and graphical representation were performed using SPSS software version 29.0.2.0 (SPSS Inc., Chicago, Illinois, USA) and GraphPad Prism version 10.0.0 (GraphPad Software, San Diego, California, USA). Statistical significance was set at *p* < 0.05 and all tests were two-sided.

## 3. Results

### 3.1. Demographics

A total of 305 potential subjects with STGD1 were identified with the data exploration tool ‘SlicerDicer'. After applying inclusion and exclusion criteria, 53 eyes of 53 patients (mean age 32 ± 19 years, 60.4% female) were included in the study. Thirty-three patients were categorized as supplement takers (mean age 34 ± 20.3 years, 57.6% female) and 20 patients as nontakers (mean age 29.5 ± 16.6 years, 65% female).  Table 1 and Table S1 summarize the demographics and clinical features of the two groups, and Tables 2 and 3 provide the percentage use and individual use for each dietary supplement.

### 3.2. Clinical Findings

In supplement takers, the mean BCVA at baseline and at follow-up was 54.9 ± 22.1 ETDRS letters (Snellen equivalent, 20/80) and 50 ± 20.7 ETDRS letters (Snellen equivalent, 20/100) (*p*=0.02), respectively, with a mean ETDRS letter score visual acuity reduction during the follow-up interval of 4.8 ± 11.2. In nontakers, the mean BCVA was 53.3 ± 16.6 ETDRS letters (Snellen equivalent, 20/80) at baseline and 44.5 ± 21 ETDRS letters (Snellen equivalent, 20/125) at follow-up (*p* < 0.01). The mean ETDRS letter score visual acuity reduction during the follow-up interval was 8.8 ± 8.4. The mean observation period for follow-up was 2 years (range, 0.5–5.6 years) in supplement takers and 4.5 years (range, 1–10.3 years) in nontakers.

### 3.3. Imaging Findings

For each patient, clinical imaging data were available for a subset of visits (a total of 174 eye visits of 53 eyes). At baseline, yellow-white pisciform flecks were observed in 75.8% of supplement takers and 85% of nontakers (*p*=0.42). Unifocal lesions were present in 84.8% of supplement takers and 80% of nontakers (*p*=0.7). Fovea-involving atrophy was detected in 84.9% of supplement takers and 85% of nontakers (*p*=0.99). Peripapillary atrophy was observed in 18.2% of supplement takers and 30% of nontakers (*p*=0.32). Increased FAF signal at the edge of a lesion DAF was present in 27.3% of supplement takers and 45% of nontakers (*p*=0.2). The baseline imaging findings are summarized in Table 4.

**Figure 2:**
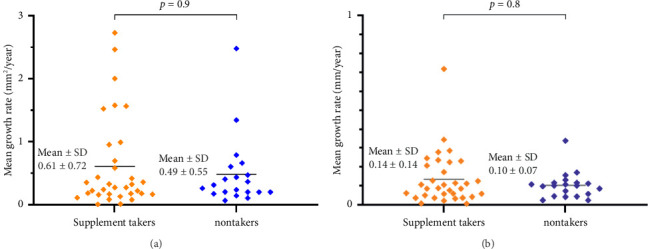
Scatter plots. (a) Scatter plot showing mean growth rates for supplement takers and nontakers using standard area measurements. (b) Scatter plot illustrating mean growth rates for supplement takers and nontakers using the square root transformation of standard area measurements. *p* values were derived from Mann–Whitney *U* tests. Significant at *p* < 0.05.

The mean lesion size at baseline was 4.681 ± 5.98 mm^2^ in supplement takers and 3.808 ± 3.60 mm^2^ in nontakers (*p*=0.9). Mean effective square root lesion size at baseline, determined as the square root of DAF lesions, was 1.879 ± 1.09 mm in supplement takers and 1.770 ± 0.84 mm in nontakers (*p*=0.99). The mean growth rates of DAF were 0.61 ± 0.72 mm^2^/year for supplement takers and 0.49 ± 0.55 mm^2^/year for nontakers (*p*=0.9) (Figure 2(a)). Estimated yearly growth rates of mean effective square root lesion size was 0.14 ± 0.14 mm/year for supplement takers and 0.10 ± 0.07 mm/year for nontakers (*p*=0.8) (Figure 2(b)).

**Figure 3:**
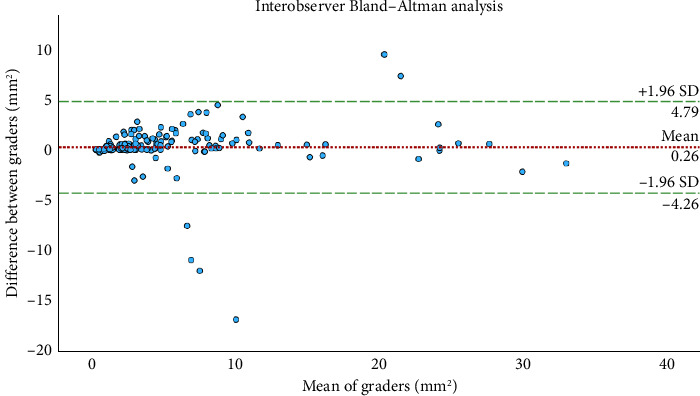
Bland–Altman plot. No systematic difference between the values obtained by the two graders for the measured atrophic lesion area was identified. The intraclass correlation coefficient for atrophy was 0.96. Measurements differed by > 0.3 mm^2^ in 113 visits, therefore requiring senior grading and arbitration. Average difference between graders ± 1.96 standard deviation (dashed lines).

The average measure intraclass correlation coefficient was calculated as 0.96 (95% CI, 0.95–0.97) for interobserver agreement, representing excellent agreement. The Bland–Altman analysis was also performed and no bias was found for interobserver measurements (Figure 3).

## 4. Discussion

We present findings of a retrospective cohort study of patients with genetically confirmed STGD1 including qualitative and quantitative FAF assessment. In this study, no favorable effect of dietary antioxidant intake on the rate of progression of atrophic lesions in STGD1 by means of FAF imaging was identified. The yearly growth rates of atrophic lesions described herein are within the range of rates of progression reported in the literature [18, 20–22], and the semiautomated method used in this study showed excellent intergrader agreement in evaluating areas of DAF. Qualitative FAF features were similar in both the supplement taker and nontaker subgroups. In addition, there was a significant reduction in mean BCVA in both supplement takers and nontakers during the respective follow-up intervals.

To our knowledge, this is the first study evaluating the efficacy of a potential antioxidant treatment in STGD1 characterized by atrophic lesions by means of FAF. The hypothesis that antioxidants may slow disease progression is warranted given the promising results of previous studies [11, 14]. Lipofuscin fluorophores, in particular A2E, represent key contributors in the pathogenesis of retinal degenerations such as STGD1 [30]. Photooxidation of A2E by highly phototoxic blue light leads to the generation of reactive oxygen species which can result in retinal cell death. The carotenoids lutein and zeaxanthin in particular are believed to protect the retina from photooxidation by filtering blue light and by acting as antioxidants [12]. Zeaxanthin is present at high concentrations at the center of the fovea, while lutein is more diffusely distributed at much lower concentrations [31]. These macular carotenoids are also present but in lower concentrations in the RPE, where they may also protect against A2E-mediated photooxidation associated with inherited retinal degenerations [32]. Macular carotenoids are derived entirely from the diet, and supplementation is believed to be beneficial for retinal health.

The Lutein Nutrition effects measured by autofluorescence (LUNA) study investigated macular pigment optical density (MPOD) measured by FAF and the serum concentrations of its constituent carotenoids in response to supplemental lutein and zeaxanthin and co-antioxidants in patients mostly affected by dry age-related macular degeneration. Supplementation with lutein and zeaxanthin, combined with co-antioxidants, resulted in an increase of MPOD in most subjects [33]. Interestingly, the authors were unable to demonstrate an increase of macular pigment in a substantial proportion of supplement takers over the study period, despite the expected and observed rises in serum concentrations of lutein and zeaxanthin. Subjects with low baseline MPOD were more likely to exhibit a dramatic rise in MPOD, or to exhibit no rise in MPOD, in response to supplements, than subjects with medium to high baseline MPOD values. Importantly, the study suggested that saturable mechanisms may govern the retinal uptake and/or stabilization of lutein and zeaxanthin [33]. Therefore, even with the proper flow of antioxidants to the eye, a limit may exist to the amount of carotenoids certain retinas can absorb. It has also been reported that MPOD in STGD1 patients is significantly lower than in healthy human subjects [34]. Based on the results and conclusions of the LUNA study, it could be hypothesized that saturable mechanisms that govern the retinal uptake of antioxidants, which appears to vary between individuals, may exist in patients with inherited retinal degenerations such as STGD1.

Many ongoing and upcoming clinical trials for STGD1 create the need for accurate structural and functional assessment of the disease, with noninvasive, repeatable, accurate, and sensitive assessments [35]. Our study provides a reliable assessment of yearly progression rates of atrophic lesions, with good agreement in measurements, something previously well documented for STGD1 [23]. More detailed structural assessments such as OCT, with ellipsoid zone width and area metrics [36], adaptive optics high-resolution photoreceptor imaging [37], and retinal functional testing such as microperimetry [38], may provide a better insight into a beneficial effect of antioxidants in patients with inherited retinal degenerations, as it has been suggested that photoreceptor degeneration may precede RPE loss in STGD1, or that functional RPE loss precedes the structural loss of RPE leading to photoreceptor loss before structural damage becomes apparent on FAF [14, 23].

Finally, our study shows that there was a trend to a more pronounced visual acuity reduction in nontakers compared to supplement takers during the follow-up intervals. This finding needs to be viewed with caution, however, since the follow-up period for nontakers was more than twice as long compared with supplement takers.

Our study has limitations, namely the retrospective design, the small sample size, the cohort heterogeneity in terms of both age and disease duration, and the variable follow-up duration. Furthermore, the lack of adjustment of supplement intake based on the patient's bodyweight may have affected the consistency of the effect of antioxidant supplements on the rate of progression of atrophic lesions [12]. In addition, when assessing the yearly growth rates of atrophic lesions, the effects of lesion perimeter and distance of the lesions from the fovea were not accounted for in our analysis [39, 40]. Due to the retrospective nature of our study, we could not assess serum concentrations of antioxidants and therefore could not determine the number of patients with a possibly limited treatment response due to a lower amount of antioxidant levels reaching the retina or the compliance to treatment.

In conclusion, we investigated in a molecularly confirmed STGD1 cohort, in masked manner and with excellent interobserver agreement the effect of antioxidant supplementation in disease progression. Supplementation with dietary antioxidants did not result in a slower progression rate of DAF lesions in STGD1. Further studies are needed to assess whether antioxidants could be an effective therapeutic intervention against STGD1.

## Figures and Tables

**Table 1:** Baseline characteristics of included participants.

	Cohort	Supplement takers	Nontakers
No. patients (*n*)	53	33	20
Age, yr (mean ± SD)	32 ± 19	34 ± 20	30 ± 17
Gender, M/F (*n*)	21/32	14/19	7/13
Ethnicity, *n* (%)			
African American	5 (9.4)	3 (9.1)	2 (10)
Asian	3 (5.7)	3 (9.1)	0 (0)
Hispanic	1 (1.9)	0 (0)	1 (5)
Native American	1 (1.9)	0 (0)	1 (5)
White	43 (81.1)	27 (81.8)	16 (80)

Abbreviations: Yr = years, M/F = male/female.

**Table 2:** Dietary antioxidants use.

Antioxidant	Patients, *n* = (%)
Lutein	32 (97)
Zeaxanthin	32 (97)
Saffron	30 (91)
N-Acetylcysteine (NAC)	32 (97)

Abbreviation: NAC = N-acetylcysteine.

**Table 3:** Dietary antioxidants use per patient.

Patient	Bodyweight (kg)	Duration (years)	Lutein (mg/day)	Zeaxanthin (mg/day)	Saffron (mg/day)	NAC (mg/day)
1	122	2	20	20	88.5	1200
2	68	2.6	20	20	15	1200
3	74.6	0.5	20	20	15	1200
4	63.9	0.5	20	20	15	600
5	30	3	20	10	15	1200
6	60.7	5	20	20	15	600
7	87.1	1	20	20	15	600
8	133.8	0.5	20	20	88.5	1200
9	90.7	1	20	20	15	600
10	68.1	1.2	20	20	—	—
11	80.7	2	20	20	15	1200
12	56.7	3.9	10	10	15	600
13	62.1	4	10	10	15	600
14	99.8	2	20	20	—	1200
15	74.8	1.1	20	20	15	600
16	49.9	5.6	20	20	15	600
17	117.2	1.2	20	20	15	600
18	71.7	1.1	20	20	15	600
19	69.9	1.9	20	20	88.5	1200
20	56.7	3.3	20	20	15	600
21	71.7	1	20	20	88.5	1200
22	114.4	2.2	—	20	—	1200
23	54.4	4.8	20	20	15	600
24	67.1	2	20	—	15	1200
25	74.8	1.6	20	20	15	1200
26	65.8	1.3	20	20	15	1200
27	22.7	1.5	20	20	15	1200
28	95.5	0.6	20	20	15	1200
29	72.6	1.3	10	10	15	1200
30	67.7	1	20	20	88.5	1200
31	67.4	2	10	10	15	1200
32	55.8	3.3	20	20	15	600
33	90.7	1.4	10	10	15	1200

Abbreviation: NAC = N-acetylcysteine.

**Table 4:** FAF characteristics at baseline for supplement takers and nontakers.

Findings	Supplement takers (*n* = 33)	Nontakers (*n* = 20)	*p* value^∗^
Atrophic lesion, *n* (%)			
Unifocal	28 (84.8)	16 (80)	0.7
Multifocal	5 (15.2)	4 (20)	
Atrophic lesion, location, *n* (%)			
Foveal	28 (84.8)	17 (85)	0.99
Nonfoveal	5 (15.2)	3 (15)	
Increased AF at lesion edge, *n* (%)			
Present	9 (27.3)	9 (45)	0.2
Absent	24 (72.7)	11 (55)	
Peripapillary atrophy, *n* (%)			
Present	6 (18.2)	6 (30)	0.32
Absent	27 (81.8)	14 (70)	
Pisciform flecks, *n* (%)			
Present	25 (75.8)	17 (85)	0.42
Absent	8 (24.2)	3 (15)	

Abbreviation: FAF = fundus autofluorescence.

^∗^ *p* values derived from chi-Square (*χ*^2^) analyses. Statistical significance was set at *p* < 0.05.

## Data Availability

The data that support the findings of this study are available from the corresponding author upon reasonable request.

## Supporting Information

Table S1: Clinical and molecular genetic findings of 53 patients with Stargardt disease.
