# Supplementary material for: Impact of Dietary Antioxidant Supplements on Atrophic Lesion Progression in Stargardt Disease: A Retrospective Observational Study
Source: J Ophthalmol. 2025 Aug 28;2025:5231278. doi: 10.1155/joph/5231278 (PMC12411062; doi:10.1155/joph/5231278)
Supplement: Supporting Information — Additional supporting information can be found online in the Supporting Information section. [file 5231278.f1.docx]

**Table S1.** **Clinical and molecular genetic findings of 53 patients with Stargardt disease**

| Patient | | Molecular Status | Gender | | Age at baseline  (years) | | Supplement  Intake | | BCVA OD  baseline  (ETDRS letters score) | | BCVA OD  final  (ETDRS  letters score) | |  |
| --- | --- | --- | --- | --- | --- | --- | --- | --- | --- | --- | --- | --- | --- |
| 1  2 | [c.4501G>T, p.E1501X]  [c.2791G>A, p.V931M]  [c.6098T>C, p.Leu2033Pro]  [c.2588G>C, p.Gly863Ala] | | | M  F | | 46  17 | | Yes  Yes | | 70  75 | | 60  75 | |
| 3 | N/A | | | M | | 31 | | Yes | | 80 | | 80 | |
| 4 | [c.4539+2001G>A]  [IVS30+2001G>A]  [c.5882G>A, p.G1961E] | | | F | | 51 | | Yes | | 35 | | 35 | |
| 5 | [c.2453G>A, p.G818E]  [c.5318C>T, p.A1773V] | | | F | | 10 | | Yes | | 25 | | 30 | |
| 6 | [c.5882G>A, p.Gly1961Glu]  [с.3261A>C, p.Glu1087Asp] | | | F | | 63 | | Yes | | 35 | | 35 | |
| 7 | [c.5461-10 T>C]  [c.3113C>T, p.Ala1038Val] | | | M | | 65 | | Yes | | 75 | | 80 | |
| 8 | [c.3259G>A, p.E1087K]  [c.6320G>A, p.R2107H] | | | F | | 53 | | Yes | | 85 | | 85 | |
| 9 | [c.5461-10 T>C]  [c.3113C>T, p.Ala1038Val] | | | M | | 47 | | Yes | | 75 | | 65 | |
| 10 | N/A | | | M | | 17 | | Yes | | 55 | | 55 | |
| 11 | [c.2894A>G, p.Asn965Ser]  [c.5882G>A, p.Gly 1961Glu] | | | F | | 72 | | Yes | | 45 | | 40 | |
| 12 | [c.1015T>G, p.W339G]  [c.4139C>T, p.P1380L] | | | F | | 14 | | Yes | | 40 | | 20 | |
| 13 | [c.6089G>A, p.R2030Q]  [c.5882G>A, p.G1961E] | | | F | | 45 | | Yes | | 60 | | 40 | |
| 14 | [c.4539+2028C>T]  [IVS30+2028C>T]  [c.161G>A, p.C54Y] | | | F | | 37 | | Yes | | 30 | | 30 | |
| 15 | [c.4539+2001G>A]  [IVS30+2001G>A]  [c.454C>T, p.Arg152Ter] | | | M | | 12 | | Yes | | 30 | | 40 | |
| 16 | [c.1622T>C, p.L541P]  [c.5882G>A, p.G1961E] | | | F | | 20 | | Yes | | 70 | | 40 | |
| 17 | N/A | | | M | | 26 | | Yes | | 45 | | 55 | |
| 18 | [c.4069G>A, p.Ala1357Thr]  [c.1919C>T, p.Pro640Leu] | | | F | | 20 | | Yes | | 45 | | 30 | |
| 19  20  21  22  23  24  25  26  27  28  29  30  31  32  33  34  35  36  37  38  39  40  41  42  43  44  45  46  47  48  49  50  51  52  53 | [c.5461-10 T>C]  [c.5882G>A, p.G1961E]  [c.2160+1G>C IVS14+1G>C]  [c.5196+1137G>A] [IVS36+1137G>A]  [c.5882G>A, p.Gly1961Glu]  [с.3261A>C, p.Glu1087Asp]  [c.3113C>T, p.A1038V]  [c.768G>T, p.V256=]  [c.6098T>C, p.Leu2033Pro]  [c.2588G>C, p.Gly863Ala]  [c.5461-10 T>C]  [c.3113C>T, p.Ala1038Val]  [c.2588G>C, p.Gly863Ala]  [c.6342G>A, p.Val2114=]  [c.1751_1753delTTAinsAT,  p.I584NfsX65]  [c.5882G>A, p.G1961E]  [c.4469G>A, p.Cys1490Tyr]  [c.1819G>A, p.Gly607Arg]  [c.5461-10 T>C]  [c.5882G>A, p.G1961E]  [c.5584+6T>C]  [IVS39+6T>C]  [c.2609C>T, p.P870L]  N/A  [c.1906C>T, p.Q636X]  [c.2588G>C, p.Gly863Ala]  [c.1015T>G, p.W339G]  [c.6218G>A, p.Gly2073Glu]  [c.5461-10 T>C]  [c.5882G>A, p.G1961E]  N/A  [c.2588G>C, p.Gly863Ala]  [c.123G>A, p.Trp41Ter]  [c.634C>T, p.Arg212Cys]  [c.5714+5G>A]  [c.3322C>T, p.Arg1108Cys]  [c.161G>A, p.Cys54Tyr]  N/A  N/A  [c.3259G>A, p.E1087K]  [c.6320G>A, p.R2107H]  N/A  [c.4577C>T, p.Thr1526Met]  [c.3210_3211dupGT, p.Ser1071CysfsTer14]  [c.5077G>A, p.Val1693lle]  [c.5714+5G>T]  [c.5882G>A, p.Gly1961Glu]  [c.853C>T, p.Gln285Ter]  [c.2300T>A, p.Val767Asp]  [c.5882G>A, p.Gly1961Glu]  [c.2894A>G, p.Asn965Ser]  [c.2588G>C, p.Gly863Ala]  [c.2297_2299deIGTG, p.G766del]  [c.5138A>G, p.Q1713R]  [c.6079C>T, p.Leu2027Phe]  [c.1015T>G, p.Trp339Gly]  N/A  [c.2894A>G, p.Asn965Ser]  [c.5882G>A, p.Gly1961Glu]  N/A  N/A  [c.4234C>T, p.Q1412*]  [c.4685T>C, p.I1562T] | | | F  F  M  F  F  M  M  F  M  F  M  F  M  M  F  M  F  F  M  F  F  F  M  M  F  M  F  F  F  M  F  F  F  M  F | | 33  15  20  63  19  33  20  23  9  85  16  28  18  57  37  22  42  45  13  61  14  25  16  11  9  47  24  22  20  19  37  52  35  60  16 | | Yes  Yes  Yes  Yes  Yes  Yes  Yes  Yes  Yes  Yes  Yes  Yes  Yes  Yes  Yes  No  No  No  No  No  No  No  No  No  No  No  No  No  No  No  No  No  No  No  No | | 70  40  70  65  75  35  65  85  25  80  45  80  75  20  5  35  70  80  40  70  40  65  45  45  45  40  80  50  40  40  30  65  85  50  50 | | 45  45  70  55  65  50  60  85  5  65  40  60  70  20  20  20  65  80  25  65  30  60  35  35  20  40  75  45  35  35  30  60  85  20  30 | |

M/F, male/female; BCVA, best-corrected visual acuity; OD, right eye; ETDRS, Early Treatment Diabetic Retinopathy Study; N/A, not available
